# Peg-Interferon Plus Ribavirin with or without Boceprevir or Telaprevir for HCV Genotype 1: A Meta-Analysis on the Role of Response Predictors

**DOI:** 10.1371/journal.pone.0094542

**Published:** 2014-04-11

**Authors:** Nicola Coppola, Mariantonietta Pisaturo, Caterina Sagnelli, Evangelista Sagnelli, Italo F. Angelillo

**Affiliations:** 1 Department of Mental Health and Public Medicine, Section of Infectious Diseases, Second University of Naples, Naples, Italy; 2 Department of Clinical and Experimental Medicine and Surgery “F. Magrassi e A. Lanzara”, Second University of Naples, Naples, Italy; 3 Department of Experimental Medicine, Second University of Naples, Naples, Italy; National Institute of Allergy and Infectious Diseases, United States of America

## Abstract

**Background & aim:**

To compare the efficacy of pegylated-interferon (Peg-IFN) α-2a or α-2b and ribavirin given as dual therapy versus triple therapy (Peg-IFN and ribavirin plus boceprevir or telaprevir) in patients with HCV-1 chronic hepatitis naïve for anti-HCV therapy or relapsers to dual therapy in relation to the presence of constitutional, clinical and virological predictors of treatment response.

**Methods:**

Included in the meta-analysis were studies meeting these criteria: original data from randomized trials on the efficacy of dual versus triple therapy in therapy-naïve patients or relapsers; at least one primary outcome clearly defined: sustained virological response in patients with or without rapid virological response (RVR), with genotype 1a or 1b, low or high HCV load, IL28-B CC or non-CC genotype, mild or severe fibrosis; odds ratio estimates of relative risk (RR) and 95% confidence intervals; English language; and published up to the end of June 2013.

**Results:**

Seven original studies met the inclusion criteria, allowing a meta-analysis on 3,652 patients. Triple therapy was more effective than dual, regardless of IL-28B genotype, HCV sub-genotype, liver fibrosis, and baseline HCV load. In 1,045 patients who achieved RVR, SVR was more frequently achieved with dual therapy (RR = 1.11; *p* = 0.002) than triple. The same results were achieved when only the therapy-naïve patients were considered.

**Conclusions:**

Triple therapy provides a significantly higher SVR rate than dual therapy, but dual therapy obtains a significantly higher SVR rate in patients with RVR. The data stress the clinical importance of a 4-week lead-in phase in direct-acting antiviral-based treatment.

## Introduction

The combination of pegylated interferon (Peg-IFN) and ribavirin, named dual therapy in the present paper, has been recommended in the international guidelines [1] as the treatment of choice for chronic hepatitis C (CHC) over the past ten years [1], [2]. This treatment provides a sustained clearance of circulating hepatitis C virus (HCV; Sustained Viral Response-SVR) in nearly half of the patients with CHC due to hepatitis C virus genotype 1 (HCV-1). The recent introduction of the direct-acting antivirals (DAAs) NS3 protease inhibitors boceprevir and telaprevir has enhanced the SVR rate in patients with HCV-1 CHC to 70-80%. Indeed, the combination of one of these protease inhibitors with Peg-IFN and ribavirin, named triple therapy in the present paper, is more effective than dual therapy for HCV-1 CHC patients naïve for anti-HCV therapy and for those who relapsed after a first course of dual therapy [3]–[6]. Triple therapy, however, is associated with serious adverse events (AE) and entails increased costs both for drugs and for the more complex health care organization needed [3]–[7].

Some constitutional, clinical and virological predictors of treatment response have been identified both for dual and triple therapy, namely the genetic polymorphisms of the interleukin (IL) 28-B gene, the different sensitivity of the HCV strains to interferon, and the extent of liver fibrosis [8]–[10]. In spite of general agreement in defining triple therapy more effective than dual therapy [11]–[16], supported by the results of two recent meta-analyses [17], [18] and studies using a Bayesian indirect treatment comparison model [19], [20], it is still a matter of debate whether the above-mentioned predictors can identify subsets of patients who could benefit more from dual than triple therapy in terms of response to treatment and/or serious adverse events.

A meta-analysis of the currently available clinical trials was undertaken to compare the overall efficacy of triple and dual therapy in patients with CHC due to HCV-1 who were therapy-naïve or relapsers to dual therapy in relation to the presence of constitutional, clinical and virological predictors of treatment response.

## Methods

The guidelines on the quality of reporting of meta-analyses have been followed throughout the design, implementation, analysis, and reporting of this meta-analysis [21]. The review protocol is available from the corresponding author.

### Study search

A comprehensive systematic literature search in the major electronic databases including MEDLINE, EMBASE, LILACS, and the Cochrane Library was conducted to locate articles on the efficacy of Peg-IFN in combination with ribavirin versus Peg-IFN, ribavirin and a DAA, telaprevir or boceprevir, in patients with HCV-1. The search, performed from January 2008 to June 2013, used both medical subject heading (MeSH) terminology and more general search terms. The following key words were used to find the studies: treatment of HCV-related chronic hepatitis, telaprevir, boceprevir, direct-acting antivirals for HCV, NS3 protease inhibitors for HCV, Peg-IFN α-2a, Peg-IFN α-2b, and ribavirin. Additionally, the reference sections of all the articles retrieved were examined and the review articles on this topic were also manually scanned to identify possible pertinent studies. Attempts were made to contact the study authors directly to include any unpublished trial results.

### Study selection

Potentially eligible articles were selected using a two-stage process. First, two of the investigators (NC and MP) independently screened the title, abstract and key words from all citations identified by the search strategy to select the relevant articles that would meet the criteria outlined below. An inclusion/exclusion form for all papers was filled out. Reasons for the exclusion of any study were recorded independently and cross-checked for agreement. Second, studies that satisfied the inclusion criteria were retrieved for full text evaluation. Disagreements regarding the relevance of specific articles prompted a second review of the titles/abstracts and were all resolved by discussion between the two authors who were not blinded to the name of the authors of the articles, the name of the journal, or the results.

Articles were considered for inclusion in the meta-analysis if: (a) the authors investigated the efficacy of conventional doses of Peg-IFN α-2a (180µg/week) or Peg-IFN α-2b (1.5µg/kg of body weight/week) plus ribavirin versus Peg-IFN α-2a or α-2b, ribavirin and conventional doses of telaprevir (750 mg three times a day) or boceprevir (800 mg three times a day) in HCV-1 chronic hepatitis patients who were therapy-naïve or relapsers to previous Peg-IFN+ribavirin treatment; (b) the authors reported data from an original study; (c) the study was a randomized trial; (d) the authors reported at least one of the primary outcomes clearly defined as SVR (undetectable HCV RNA 6 months after the end of therapy) in patients with or without a rapid virological response (RVR; HCV RNA negative after 4 weeks of treatment), with genotype 1a or 1b, low or high HCV load, IL28-B CC/non-CC genotype, mild or severe fibrosis; (e) the authors reported data allowing the calculation of the odds ratio estimates of relative risk (RR) for the effect on different outcomes of dual versus triple therapy; (f) the article was written in English; and (g) the article was published up to June 2013. The following criteria were used for study exclusion: observational study; inclusion of patients who had undergone liver transplantation; inclusion of anti-human immunodeficiency virus (HIV)-positive patients; duplicate data or duplicate publication.

### Data extraction

The two investigators (NC and MP) independently extracted information from all selected articles according to the inclusion criteria listed above by using a pre-established data extraction form. The following data were collected from each study: first author's surname, year of publication, country of the study, time of study (start date and end date), study design, characteristics of the trial participants (number, age, gender), type of DAA, type of Peg-IFNα, RRs and standard errors of these estimates. If the latter were not available, they were calculated. Any disagreement between the investigators was resolved as mentioned above. We contacted the corresponding authors of the papers considered to obtain data on the patients' outcome not reported in their publications.

### Quality assessment

Full copies of each individual trial included in the meta-analysis were independently assessed for methodological quality by two investigators (IFA and NC) using the Jadad et al. score [22]. This 5-point quality scale consists of three items with points for randomization (0–2 points), double blinding (0–2 points), and explanation of dropouts and withdrawals (0–1 point) to be assigned to each trial. A score of 1 was given for each of the points described. An additional point was assigned when the method of randomization and/or double blinding was given and was appropriate; when it was inappropriate a point was deducted. Thus, the quality scale ranged from 0 (lowest quality) to 5 (highest quality). Final scores of 0–2 were considered as low quality, whereas final scores of ≥3 were regarded as representative of studies of high quality.

### Statistical analysis

For the studies in which no estimate of relative risk was published, these estimates were calculated based on the reported numbers of participants. Risk estimates and their standard errors were calculated for each study separately. Heterogeneity among the studies included was determined by means of two separate statistical estimates. The Cochran *Q* test was used to provide a test of statistical significance to determine whether the differences in effect sizes are due to a subject-level sampling error alone or other sources. Heterogeneity was estimated by using the I^2^ statistic, defined as the proportion of total variation observed between trials attributable to differences between trials rather than to a sampling error (chance), with high values suggesting a greater possibility of heterogeneity. Values approaching zero (0%) indicated no heterogeneity observed and higher values indicated increasing heterogeneity. This was assessed using published guidelines for low (I^2^ = 25–49%), moderate (I^2^ = 50–74%) and high (I^2^≥75%) heterogeneity [23]. For both tests a threshold *p* value less than 0.1 was considered statistically significant. In the absence of heterogeneity between the studies, the pooled estimate of each study was calculated using the Mantel-Haenszel method for a fixed-effects model [24], otherwise, the random-effects model by the DerSimonian and Laird method was used [25]. Sensitivity analyses were performed to explore the influence of the quality of the study on outcomes observed by restricting the analysis to randomized controlled trials with a Jadad et al. score greater than four. An estimate of potential publication bias was made using the funnel plot, in which the standard error of log (OR) of each study was plotted against its log (OR), and an asymmetric plot suggested a possible publication bias [26]. Funnel plot asymmetry was investigated using Egger's linear regression test, a linear regression approach to measure the funnel plot asymmetry on a natural logarithm scale of ORs, and *p*<0.1 was considered representative of a statistically significant publication bias [26]. The Begg and Mazumdar adjusted rank correlation test was used to examine the association between the effects estimates and their variances [27]. Data management and all statistical analyses were performed using Stata software SE, version 10.1 (StataCorp LP, College Station, TX, USA). The statistical significance for all *p* values from 2-sided tests was defined as less than 0.05.

## Results

### Study characteristics

**Figure 1:**
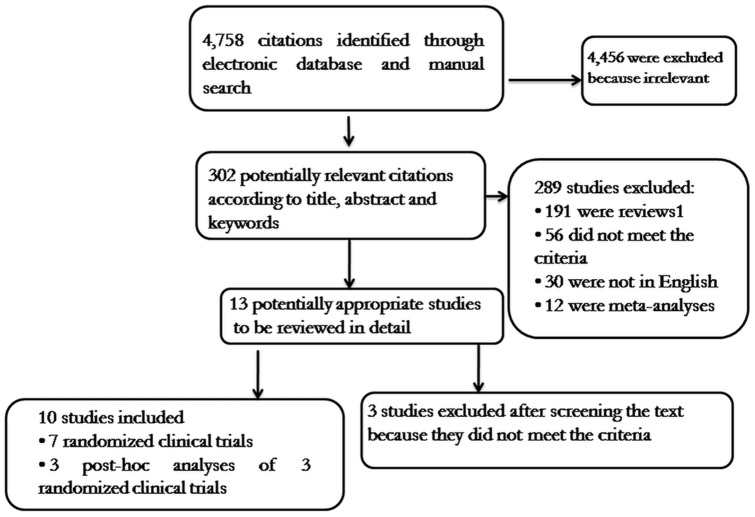
Flow chart of the published studies evaluated for inclusion in the meta-analysis.

A total of 4,759 potentially relevant titles and abstracts were identified through electronic database and manual search using the above-mentioned keywords (Figure 1). Of these, 302 citations were considered potentially relevant but only 7 [28]–[34] met the inclusion criteria (Figure 1). Three post-hoc analyses [35]–[37] were also included for a more extensive knowledge of the original studies: the Poordad study [35] for the IL28-B data of the Poordad [29] and Bacon studies [30]; the Bruno study [36] for the IL28-B data of the Poordad [29] and Bacon studies [30]; the Pol study [37] for the fibrosis data of the Zeuzem study [32].

The main characteristics of the seven randomized controlled trials and of the patients enrolled are presented in Tables 1 and 2. The studies were published between 2010 and 2013 and sample sizes ranged from 145 to 1,097 patients. Four trials [28], [29], [31], [33] enrolled therapy-naïve patients and three [30], [32], [34] relapser patients to previous Peg-IFN+ribavirin treatment. Three studies experimented telaprevir [31]–[33] and four boceprevir [28]–[30], [34] as the DAAs. In all studies, the ribavirin dose was weight-based ranging from 600 to 1,400 mg. All patients treated with dual therapy received treatment for 48 weeks. The studies used different strategies in the administration of DAA treatment. A lead-in strategy (Peg-IFN+ribavirin in the first 4 weeks of treatment and then triple therapy) was used in all boceprevir studies [28]–[30], [34], with the exception of only two arms in one study [28]. A combination of Peg-IFN and ribavirin plus telaprevir was administered for 12 weeks followed by dual therapy for the subsequent 12–36 weeks in all the studies using telaprevir except two [31], [32]. In particular, a combination of Peg-IFN+ribavirin and telaprevir was administered for the first 8 weeks in one arm of one study [31] and a lead-in strategy was applied in one arm of the other study [32].

**Table 1:** General characteristics of the clinical trials included in the meta-analysis.

First Author [Reference No.]	Geographical area	Enrolment period	Type of patients	Type of DAA	Type of Peg-IFN α	No. of patients PR/PR+DAA	Contact with authors successful
Kwo [28]	North America, Europe	From 2007 to 2008	Naïve	Boceprevir	2b	104/416	No
Poordad [29]	America, Europe	2008	Naïve	Boceprevir	2b	363/734	No
Bacon [30]	North America, Europe	2008	Relapser	Boceprevir	2b	51/208	Yes
Jacobson [31]	America, Europe, Australia	Not reported	Naïve	Telaprevir	2a	361/727	No
Zeuzem [32]	America, Australia, Europe	From 2008 to 2010	Relapser	Telaprevir	2a	68/286	Yes
Kumada [33]	Japan	From 2008 to 2010	Naïve	Telaprevir	2b	63/126	No
Flamm [34]	North America, Europe	2009	Relapser	Boceprevir	2a	47/98	No

Peg-IFN α: pegylated interferon; PR: pegylated interferon plus ribavirin; DAA: direct-acting antivirals

**Table 2:** General characteristics of the patients from each clinical trial included in the meta-analysis.

First Author [Reference No.]	No. of patients, PR/PR+DAA	Age (mean) in years, PR/PR+DAA	% males, PR/PR+DAA	% of IL28-B CC patients, PR/PR+DAA	% of patients with RVR, PR/PR+DAA	% of patients without advanced fibrosis, PR/PR+DAA	% of patients with genotype 1b, PR/PR+DAA	% of patients with low HCV RNA, PR/PR+DAA
Kwo [28]	104/416	48/47	67/56	/	8/51	92/93	40/33	11/10
Poordad [29]	363/734	49/49	57/61	29/30	9/6	90/86	35/36	15/15
Bacon [30]	51/208	52.9/52.3–52.9*	72/65*	31/20	14/61	75/75	45/40	24/14
Jacobson [31]	361/727	49/49ç	58/58	34/32	9/67	80/78	42/41	23/23
Zeuzem [32]	68/286	50/51*	67/70*	23/28	3/37	56/58	46/49	18/16
Kumada [33]	63/126	55/53ç	52.4/52.4	/	/	/	100/98	71/79
Flamm [34]	47/98	53/52*	64/72*	26/24	/	70/69*	40/41*	19/25*

Peg-IFN α: pegylated interferon; PR: pegylated interferon plus ribavirin; DAA: direct-acting antivirals; RVR: rapid virological response;ç: median; *: evaluated for relapsers and non-responder patients

Advanced liver fibrosis was defined by the presence of bridging fibrosis or cirrhosis [31], [32], cirrhosis [28], or a staging score equal to or higher than 3 in the METAVIR scoring system [29], [34], [36]. The correlation between the achievement of SVR and the entity of HCV load was investigated using different cut-off values of high HCV RNA: 800,000 IU/mL [29]–[32], [36], 600,000 IU/mL [28], and 7 log_10_ IU/mL [33].

Anemia was defined as values of hemoglobin less than 10 g/dl [28], [31], [32] or less than 9.5 g/dl [29], [30], [33], [34], severe anemia as values of hemoglobin less than 8 g/dl [29], [30], [33], [34] or less than 8.5 g/dl [28], [31], [32], and neutropenia as neutrophil cells less than 750 cells/ml.

### Assessment of quality

The methodological quality data according to the Jadad scale are presented in Table 3. Of the 7 trials included, 6 were classified as having high methodological quality and scored 3 or more points, but none of the trials gained the maximum score or scored 0. The randomization procedure was reported in sufficient detail assuring its appropriateness in 6 studies, but was not reported in one. Four trials stated that they had adopted double-blind methods but did not give details. Details of dropouts were reported in all trials with rates ranging from 4.8% to 14.8%; the reasons for patient withdrawals or dropouts were given.

**Table 3:** Distribution of studies by quality scoring according to Jadad et al.

First Author [Reference No.]	Was the treatment randomly allocated?	Was the randomization procedure described and was it appropriate?	Was the trial described as double blind?	Was the method of blinding described and appropriate?	Was the number of withdrawals/dropouts in each group mentioned?	Jadad Score, Maximum Score = 5
Kwo [28]	Yes	Yes	No	-	Yes	3
Poordad [29]	Yes	Yes	Yes	No	Yes	4
Bacon [30]	Yes	Yes	No	-	Yes	3
Jacobson [31]	Yes	Yes	Yes	No	Yes by group/No drop outs	4
Zeuzem [32]	Yes	Yes	Yes	No	Yes	4
Kumada [33]	Yes	No	No	-	Yes	2
Flamm [34]	Yes	Yes	Yes	No	Yes	4

### Effects of Interventions

**Figure 2:**
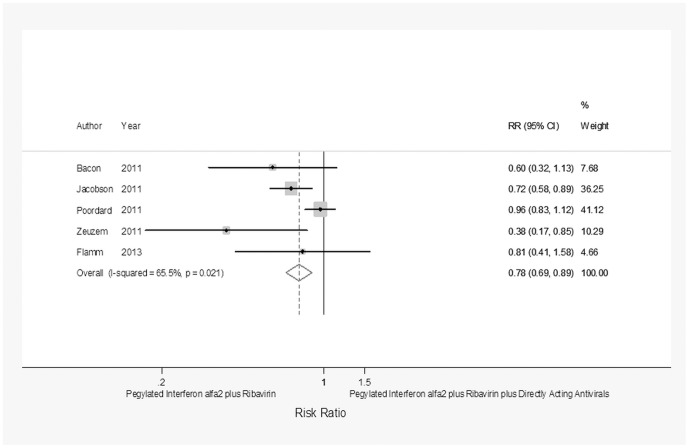
Forest plot showing the achievement of SVR in CHC patients with the IL28-B CC haplotype treated with pegylated interferon α-2 plus ribavirin or pegylated interferon α-2 plus ribavirin plus a direct-acting antiviral.

**Figure 3:**
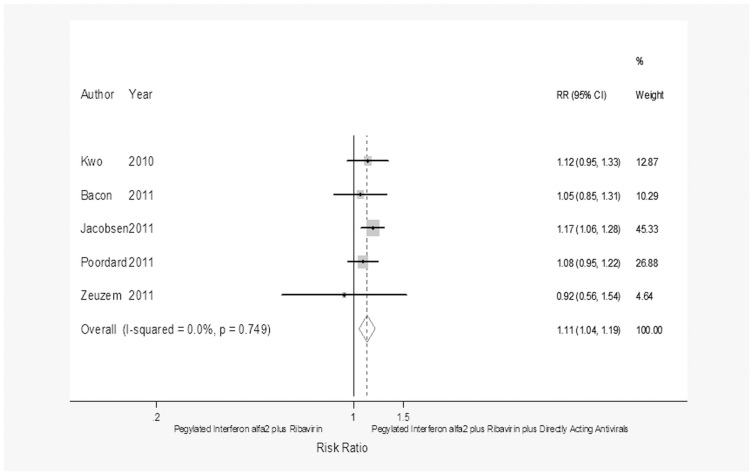
Forest plot showing the achievement of SVR in CHC patients with a rapid virological response treated with pegylated interferon α-2 plus ribavirin or pegylated interferon α-2 plus ribavirin plus a direct-acting antiviral.

**Figure 4:**
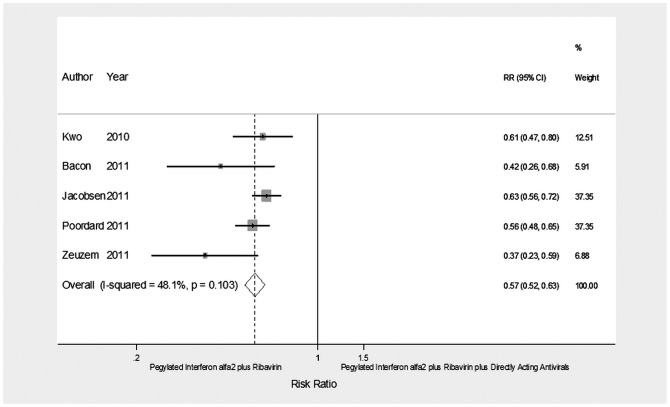
Forest plot showing the achievement of SVR in CHC patients without advanced liver fibrosis treated with pegylated interferon α-2 plus ribavirin or pegylated interferon α-2 plus ribavirin plus a direct-acting antiviral.

**Figure 5:**
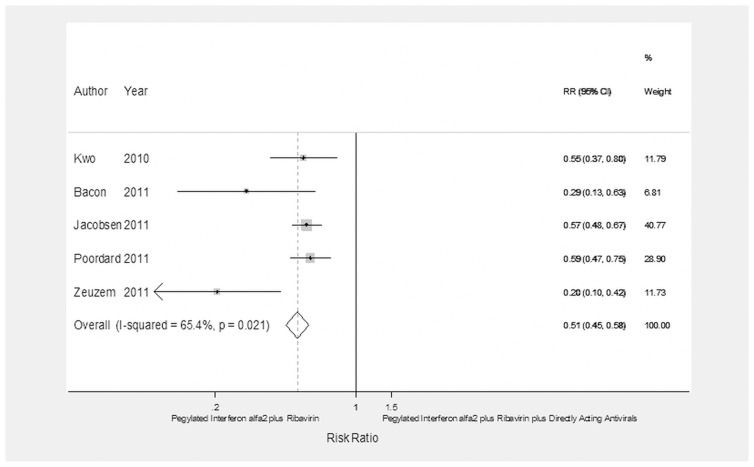
Forest plot showing the achievement of SVR in CHC patients with genotype 1b treated with pegylated interferon α-2 plus ribavirin or pegylated interferon α-2 plus ribavirin plus a direct-acting antiviral.

**Figure 6:**
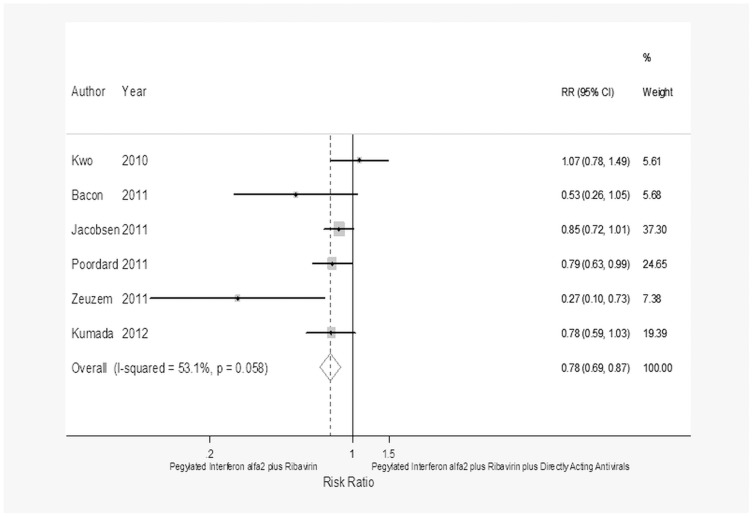
Forest plot showing the achievement of SVR in CHC patients with low baseline HCV RNA treated with pegylated interferon α-2 plus ribavirin or pegylated interferon α-2 plus ribavirin plus a direct-acting antiviral.

Table 4 and Figures 2, 3, 4, 5, and 6 show the efficacy of dual versus triple therapy according to the different outcomes. The rate of SVR according to the IL28-B genotype was assessed in 5 studies with a total of 487 patients with IL28-B CC and 1,196 with IL28-B CT/TT genotypes. The analysis of the pooled data showed that dual therapy less frequently than triple therapy achieved an SVR both in patients with IL28-B CC genotype (RR = 0.78; 95% CI = 0.69–0.89, *p*<0.0001) and those with IL28-B CT/TT (RR = 0.4; 95% CI = 0.33–0.47, *p*<0.0001).

**Table 4:** Meta-analysis data on the achievement of SVR and tolerability with pegylated interferon α plus ribavirin or pegylated interferon α, ribavirin and a direct-acting antiviral in patients with chronic hepatitis C due to HCV genotype 1.

SVR in patients with	N° of studies	N° of patients PR/PR+DAA	N° and (%) of events PR/PR+DAA	RR (efficacy)	95% CI (efficacy)	*p*	Heterogeneity test (Q;*p*;I^2^,%)
IL28-B CC	5 ^29–32,34^	150/337	99(66)/283(84)	0.78	0.69–0.89	<0.0001	11.58;0.021;65.5
IL28-B CT or TT	5^29–32,34^	347/849	92(26.5)/569(67)	0.4	0.33–0.47	<0.0001	11.03;0.026;63.7
RVR	5^28–32^	80/965	77(96)/810(84)	1.11	1.04–1.19	0.002	1.93;0.75;0.0
No RVR	5^28–32^	890/1,356	286(32)/831(61)	0.56	0.5–0.62	<0.0001	24.85;<0.0001;83.9
No advanced fibrosis	5^28–32^	788/1,911	322(41)/1,360(71)	0.57	0.52–0.63	<0.0001	7.70;0.1;48.1
Advanced fibrosis	5 ^28–32^	145/422	41(28)/271(64)	0.45	0.34–0.59	<0.0001	11.32;0.023;64.7
Genotype 1a	5^28–32^	500/1,287	183(37)/769(60)	0.55	0.49–0.62	<0.0001	3.82;0.43;0.0
Genotype 1b	5^28–32^	373/898	152(41)/700(78)	0.51	0.45–0.58	<0.0001	11.58;0.021;65.4
Low HCV RNA	6^28–32^	217/437	135(62.2)/389(79)	0.78	0.69–0.87	<0.0001	10.67;0.058;53.1
High HCV RNA	6^28–32^	793/2,005	261(33)/1,355(67.5)	0.54	0.49–0.6	<0.0001	21.79;0.001;77.1
Patients with							
Discontinuation for AE	7^28–34^	1,170/2,987	122(10)/471(16)	0.67	0.55–0.81	<0.0001	20.77;0.002;71.1
Severe AE	6^28–32,34^	1,107/2,861	76(0.6)/297(10)	0.65	0.51–0.83	0.001	2.98;0.7;0.0
Anemia	7^28–34^	1,170/2,987	202(17)/1,124(38)	0.47	0.41–0.54	<0.0001	5.89;0.44;0.0
Severe anemia	7^28–34^	1,170/2,987	23(0.2)/208(0.7)	0.3	0.2–0.46	<0.0001	5.05;0.54;0.0
Neutropenia	5^28–30,32,34^	746/2,137	132(18)/621(29)	0.59	0.5–0.7	<0.0001	3.12;0.54;0.0

SVR: sustained virological response; PR: pegylated interferon plus ribavirin; DAA: direct-acting antivirals; IL28-B: interleukin 28B; RVR: rapid virological response; AE: adverse events.

As regards the achievement of RVR, dual therapy achieved an SVR more frequently than triple therapy in the 1,045 patients who achieved an RVR (RR = 1.11; 95% CI = 1.04–1.19, *p* = 0.002) whereas dual therapy attained an SVR less frequently than triple therapy (RR = 0.56; 95% CI = 0.5–0.62, *p*<0.0001) in the 2,246 patients who did not achieve an RVR.

Moreover, SVR was less frequently observed in patients treated with dual therapy than in those receiving triple therapy in the five studies on 2,699 patients with advanced liver fibrosis (RR = 0.45; 95% CI = 0.34–0.59, *p*<0.0001) and on 567 patients without advanced liver fibrosis (RR =  0.57; 95% CI = 052–0.63, *p*<0.0001).

As regards the HCV-1 subgenotype, dual therapy less frequently than triple therapy achieved SVR both in 1,797 patients with HCV-1a subgenotype (RR = 0.55; 95% CI = 0.49–0.62, *p*<0.0001) and in 1,271 with HCV-1b (RR = 0.51; 95% CI = 0.45–0.58, *p*<0.0001).

The frequency of SVR in relation to HCV load was investigated pooling six studies with 709 patients with a low baseline HCV load and 2,798 patients with high baseline HCV load, and in both groups dual therapy achieved an SVR less frequently than triple therapy (RR = 0.78; 95% CI = 0.69–0.87, *p*<0.0001; RR = 0.54; 95% CI = 0.49–0.6, *p*<0.0001, respectively).

Pooling the 4 studies including only therapy-naïve patients, the results were similar for all outcomes to those achieved for therapy-naïve and relapser patients pooled together.

In the 7 trials included in the meta-analysis, adverse events (AE) leading to the discontinuation of treatment were observed in 121 (11.4%) of the 1,057 patients treated with dual therapy and in 470 (18.1%) of the 2,595 receiving triple therapy, i.e., a 33% lower occurrence in those with dual therapy (RR = 0.67; 95% CI = 0.55–0.81, *p*<0.0001). Similar results were achieved for the rate of serious AE, anemia, severe anemia and neutropenia. Dual therapy, compared to triple therapy, was associated with a decreased occurrence of these events ranging from 35% to 70% (Table 4). The sensitivity analysis showed that when the low-quality study was not included in this meta-analysis, the results were consistent and similar to the overall pooled estimates.

There was evidence of heterogeneity between studies only for the non-achievement of RVR and high HCV RNA at baseline. Moderate heterogeneity was observed for IL28-B CC genotype and HCV genotype 1a, whereas the other subgroups showed low heterogeneity. A funnel plot was generated for each analysis. According to the funnel plots of the logarithm of estimated OR against the standard error, evidence of publication bias among studies was observed only for the discontinuation of treatment (Egger's test *p* = 0.005), and no other significant publication bias was observed. Therefore, publication bias, if any, had no effect on the results of this meta-analysis.

## Discussion

This meta-analysis is the most comprehensive review of the literature evaluating the efficacy of dual therapy with Peg-IFN α-2a or α-2b plus ribavirin versus triple therapy with Peg-IFN+ribavirin and a first-generation NS3 protease inhibitor, telaprevir or boceprevir, in treating therapy-naïve or relapser anti-HIV-negative patients with HCV-1 CHC. The two above-mentioned meta-analyses demonstrated that triple therapy more frequently achieved SVR both in therapy-naïve and in previous non-responder patients [17], [18], although with a significantly greater incidence of AE, mainly anemia and skin rash [17], [18], [38]. However, it should be noted that these meta-analyses did not investigate the role of the pre- or on-therapy predictors of response to treatments

An RVR was less frequently achieved by patients treated with dual therapy (9-16%) than in those with triple therapy (37–65%) [28], [29], [31], [32], but patients who achieved RVR under dual therapy achieved an SVR more frequently than those with an RVR under triple therapy. These differences are not easily explained, but it may be hypothesized that patients who develop an RVR under a less effective treatment are more frequently prone to maintaining this favorable response and to obtaining SVR under the same treatment than those with an RVR achieved with a stronger treatment, probably because of a more frequent occurrence of AE with triple therapy. It seems reasonable to conclude on this point that RVR, although less frequent in patients treated with the less effective treatment, may be a more reliable predictor of HCV eradication.

The efficacy of triple therapy in therapy-naïve and relapser patients with HCV-1 chronic hepatitis was greater than that of dual therapy regardless of the IL-28B genotype, the severity of liver fibrosis, HCV sub-genotype, and HCV load at the baseline, thus indicating that these factors do not affect the different efficacy between dual and triple therapy in these patients. With regard to the HCV load at the baseline, however, a sub-analysis performed on therapy-naïve patients showed that triple therapy was more effective than dual therapy in those with a high HCV load, whereas no difference in the SVR rate was found in patients with a low HCV load. The latter observation seems to be of clinical value even if achieved in patients from 4 trials using cut-off values to define a low or high viral load ranging from 600,000 to 7 log_10_ IU/ml. This may introduce a bias and is likely to contribute to the underestimation of the true burden of effect. The low number of relapser patients in this meta-analysis did not allow us to perform a similar sub-analysis in this subset of patients.

The differences between the two treatments in the rates of AE leading to treatment discontinuation, anemia, severe anemia, and neutropenia were also examined. AE had a strong clinical impact on both double and triple therapy, since more than 10% of patients discontinued treatment. A significantly greater frequency of serious AE and of AE leading to treatment discontinuation was recorded in triple than in dual therapy, reducing at least in part the advantages offered by triple therapy. To this regard it should also be noted that none of the studies included was designed to evaluate AE and, consequently, the incidence of AE may have been underestimated.

It is encouraging that the methodological quality of each trial included in the meta-analysis, as indicated by the Jadad scale, was high (a score of 3 or more points) in 6 of the 7 trials, but none gained the maximum score. All trials claimed to have adopted randomization, and only one trial did not give any information on the randomization method. None of the seven trials described the methods of treatment allocation concealment. Thus, whether randomization was effectively conducted in these trials is doubtful. Inappropriate randomization or allocation concealment can lead to a selection bias. Four out of the seven trials were double-blinded, although the method was not described. Dropout cases were reported in all seven trials and the low withdrawal rates may indicate the quality of the data. A selection bias, performance bias, and attribution bias may overestimate the efficacy of treatment.

Although this meta-analysis provides useful information, some potential limitations should be addressed. First, heterogeneity among the studies included is a crucial problem, and inappropriate management may induce misleading statistical inference. Heterogeneity between the studies was observed in the present meta-analysis, which suggests that the study designs contributed to this heterogeneity to some extent. There were marked differences in sample size, patient's source, and stratification criteria and these may result in statistical biases. All factors that may interfere with statistical inference have been carefully considered in this meta-analysis. Although two of the meta-analyses performed contained a small number of studies, funnel plots were also made to assess publication bias, and no obvious asymmetry was observed. Second, trials on both therapy-naïve and relapser patients were included and telaprevir and boceprevir, as the DAA, were investigated together. However, therapy-naive and patients who relapsed after a first course of dual therapy had a similar probability of SVR with triple therapy [3]–[6]. Patients treated with boceprevir and telaprevir in triple therapy were analyzed together because of the small number of studies available and because they are both first-generation NS3 protease inhibitors that have demonstrated a similar antiviral effect. Third, the possibility of a publication bias needs to be borne in mind, particularly in a meta-analysis based only on published studies, because “positive” studies are more likely to be submitted and published than “negative” studies, and so it cannot be ruled out that a publication bias, such as the lack of published studies with inconclusive results, may have at least a moderate impact on the results. However, because this is a new treatment, it is improbable that “negative” studies exist. Fourth, the literature search was conducted by searching multiple electronic databases, reference lists of retrieved manuscripts and reviews of experts in this field and was restricted to articles published in the English language. However, a manual search did not reveal any articles published in languages other than English. In addition, an attempt was made to avoid publication bias by seeking out clinical trial data that had not been published. Fifth, six of the trials were funded by the pharmaceutical industry and although we tried, but were unable, to include unpublished data, we cannot avoid a sponsorship bias, and our results might therefore overestimate the effect. Unfortunately, eliminating both sources of bias simultaneously is difficult, if not impossible. Although clinical trials now need to be registered in advance to be published in major medical journals, there is no requirement that the results be submitted for publication, and many failed clinical trials or clinical trials with negative results go unpublished. Even with these limitations, the clinical trials provide useful evidence regarding the efficacy of triple and dual therapy in patients with CHC due to HCV-1.

This meta-analysis on HCV-1 therapy-naïve or relapser patients found a higher SVR rate in those treated with triple therapy than those with dual therapy, regardless of IL-28B genotype, liver fibrosis, baseline HCV load, and HCV sub-genotype. The achievement of RVR identified patients with a high rate of SVR in both treatment schedules, a rate even higher in patients treated with dual therapy. Considering also the high cost and the high rate of AE of boceprevir and telaprevir, this meta-analysis emphasizes the clinical importance, for patients with HCV-1 CHC who are anti-HIV-negative and therapy-naïve or relapsers to previous dual therapy, of a 4-week lead-in phase with dual therapy followed by the addition of a first-generation DAA for patients who do not achieve an RVR.

In conclusion, the data from the present meta-analysis suggest that patients with HCV-1 CHC who are therapy-naïve or relapsers to previous dual therapy should receive a 4-week lead-in phase with Peg-IFN/ribavirin, followed by dual therapy for patients with RVR and triple therapy with a first generation DAA only for patients who do not achieve an RVR. The treatment of chronic HCV will undoubtedly change in the next few years, since new drugs have recently been approved, others are close to approval, and several others are under investigation. Safer and more effective combinations of protease inhibitors or nucleoside analogues with Peg-IFN and ribavirin will gradually be available worldwide, while awaiting the forthcoming Peg-IFN-free regimens [39]–[42]. This meta-analysis does, however, provide reliable guidance also for combination therapies with Peg-IFN/ribavirin plus a second- or third-generation DAA, since early identification of patients with a predictable SVR will allow equally effective and less expensive treatment regimens to be administered.

It is doubtful whether the high prognostic value of the RVR highlighted by this meta-analysis will have much relevance to the new Peg-IFN/ribavirin-free regimens. However, the high cost of the new generation protease inhibitors and nucleoside analogues and, consequently, of the Interferon-free treatments, will presumably limit the access to these new treatments in several countries, where it is foreseeable that combinations with Peg-IFN, ribavirin and first generation protease inhibitors will be used for years to come.

## Supporting Information

Checklist S1 **PRISMA Checklist.**
(DOC)
